# Supplementary material for: Splice-Junction-Based Mapping of Alternative Isoforms in the Human Proteome
Source: Cell Rep. Author manuscript; Available in PMC 2020 Jan 15. (PMC6961840; doi:10.1016/j.celrep.2019.11.026)
Supplement: 2 [file NIHMS1546469-supplement-2.zip › DF1/PXD010154/Ovary/Ovary_14_LYPLAL1_IIYPTAPPSYTVGPSFAR.pdf]

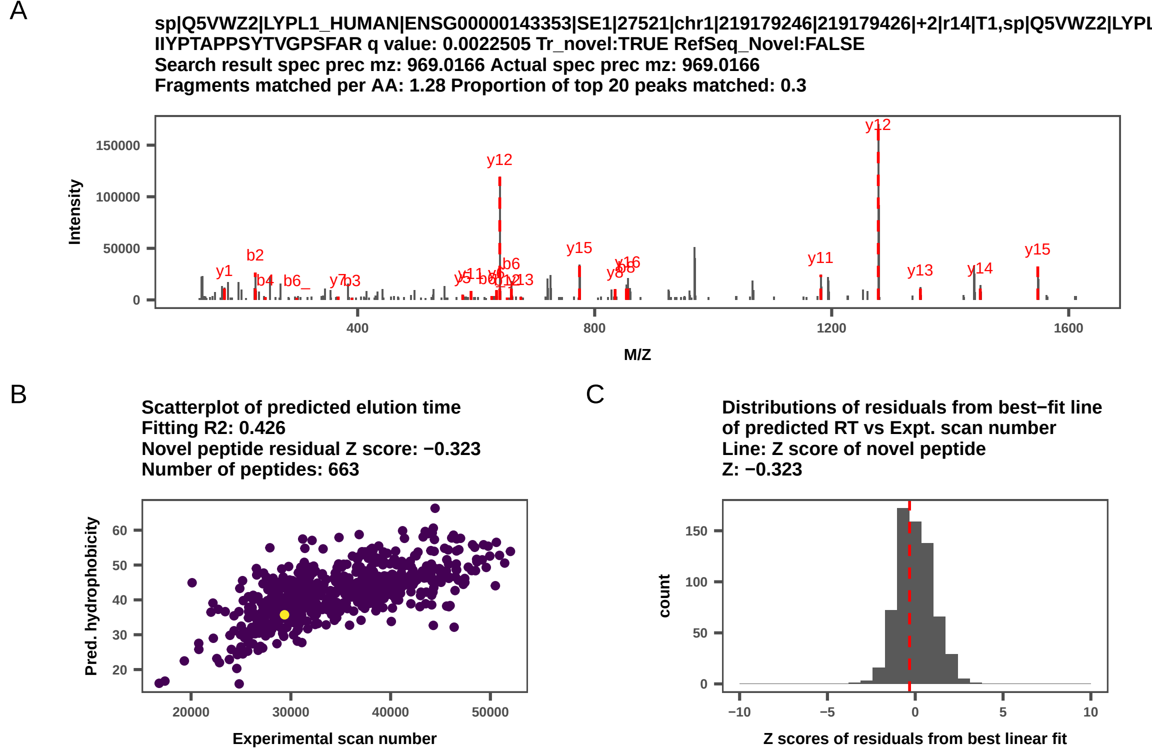
A
sp|Q5VWZ2|LYPL1_HUMAN|ENSG00000143353|SE1|27521|chr1|219179246|219179426|+2|r14|T1,sp|Q5VWZ2|LYPL
IIYPTAPPSYTVGPSFAR q value: 0.0022505 Tr_novel:TRUE RefSeq_Novel:FALSE
Search result spec prec mz: 969.0166 Actual spec prec mz: 969.0166
Fragments matched per AA: 1.28 Proportion of top 20 peaks matched: 0.3
Intensity
150000
100000
50000
0
400
800
1200
1600
M/Z
y1
b2
b4
b6_
y7
b3
y12
y11
b6
y15
y16
y11
y12
y13
y14
y15
B
Scatterplot of predicted elution time
Fitting R2: 0.426
Novel peptide residual Z score: −0.323
Number of peptides: 663
Pred. hydrophobicity
60
50
40
30
20
20000
30000
40000
50000
Experimental scan number
C
Distributions of residuals from best−fit line
of predicted RT vs Expt. scan number
Line: Z score of novel peptide
Z: −0.323
count
150
100
50
0
−10
−5
0
5
10
Z scores of residuals from best linear fit
